# What Is Constructionism in Psychiatry? From Social Causes to Psychiatric Classification

**DOI:** 10.3389/fpsyt.2016.00057

**Published:** 2016-04-18

**Authors:** Raphael van Riel

**Affiliations:** ^1^Philosophy, Philipps-University Marburg, Marburg, Germany; ^2^Philosophy, University Duisburg-Essen, Essen, Germany

**Keywords:** philosophy of science, explanation in psychology, metaphysics, social construction, psychiatric classification

## Abstract

It is common to note that social environment and cultural formation shape mental disorders. The details of this claim are, however, not well understood. The paper takes a look at the claim that culture has an impact on psychiatry from the perspective of metaphysics and the philosophy of science. Its aim is to offer, in a general fashion, partial explications of some significant versions of the thesis that culture and social environment shape mental disorders and to highlight some of the consequences social constructionism about psychiatry has for psychiatric explanation. In particular, it will be argued that the alleged dependence of facts about particular mental disorders and about the second order property of being a mental disorder on social facts amounts to a robust form of constructivism, whereas the view that clinician–patient interaction is influenced by cultural facts is perfectly compatible with an anti-constructivist stance.

## Introduction

It is common to note that social environment and cultural formation shape mental disorders. For instance, the Surgeon General David Satcher states in the preface to *Mental Health: Culture, Race, and Ethnicity* (Supplement) that

[t]he cultures from which people hail affect all aspects of mental health and illness, including the types of stresses they confront, whether they seek help, what types of help they seek, what symptoms and concerns they bring to clinical attention, and what types of coping styles and social supports they possess. Likewise, the cultures of clinicians and service systems influence the nature of mental health services. [(1): preface]

The details of the claim that social or cultural facts or events have a significant or systematic impact on mental disorders are, however, not well understood. Some construe it as a purely epistemological claim (2), on other occasions it is mentioned without any further explication. For instance, in the supplement *Mental Health: Culture, Race, and Ethnicity* just quoted, it is stated that

[c]ultural differences in the expression and reporting of distress are well established among American Indians and Alaska Natives. These often compromise the ability of assessment tools to capture the key signs and symptoms of mental illness […] Words such as “depressed” and “anxious” are absent from some American Indian and Alaska Native languages […]. Other research has demonstrated that certain DSM diagnoses, such as major depressive disorder, do not correspond directly to the categories of illness recognized by some American Indians. Thus, evaluating the need for mental health care among American Indians and Alaska Natives requires careful clinical inquiry that attends closely to culture. [(1): chapter 4.]

We will turn to similar examples below. Typically, such claims are regarded as articulating versions of *social constructivism*, as opposed to objectivism; authors quickly move from the idea that sociocultural environment has an impact on mental disorders to constructivist rhetoric. The paper takes a look at the claim that culture has an impact on psychiatry from the perspective of metaphysics and the philosophy of science. Its aim is to offer, in a general fashion, partial explications of versions of social constructivism about mental disorders and to show which claims regarding a sociocultural influence on mental disorders amount to social constructivism, and which claims do not. In particular, I will discuss constructivist claims about mental disorders themselves, such as posttraumatic stress disorder (PTSD), about the subjective experience, the phenomenology, and symptoms that are indicative of a disorder, about the second order property of being a mental disorder, instantiated by, for instance, PTSD or autism, and about constructivism about aspects of the clinician–patient interaction. It will turn out that social constructivist rhetoric, in many cases, does not amount to social constructivism, properly construed.^1^

Social constructivism in psychiatry can be tentatively characterized by contrasting it with what one may want to call *radical objectivism* (a view I will use for illustrative purposes only).^2^ According to radical objectivism about psychiatry, types of mental disorders, and the type *mental disorder* itself, are just like the types the natural sciences deal with, in that they are in some sense *explanatorily independent* of social facts or events.^3^ Neither do facts about mental disorders have to be explained in terms of underlying social facts nor should the occurrence of mental disorders be explained by reference to social causes, in a sense to be specified.^4^ This is what the dispute between objectivism and constructivism is about. Compare events of evaporation of water and what they, as such, depend on with events of elections and what elections, as such, depend on. Neither do explanations of events of *evaporation of water* in physics or chemistry cite social causes nor is the evaporation of water, in these sciences, explained in terms of underlying social facts. Although the evaporation of water in the ocean may depend on social facts, namely, the social causes of global warming, in an explanation of what evaporation of water *is*, we should not cite the social causes of actual evaporation of water. By contrast, it seems reasonable to assume that *elections* are to be explained in terms of social causes, such as joint decisions to vote, or decisions to hold an election by individuals or groups who have a certain social status within a society. Moreover, unlike facts about the evaporation of water, the fact that the election takes place, facts about how it develops, etc., explanatorily depend on other social facts – facts about actions of individuals that count, in the relevant context, as votings. Social constructivism about psychiatry assumes that facts about mental disorders are, with respect to what they, as such, explanatorily depend on, a bit-like facts about elections. Radical objectivism assumes that facts about mental disorders are, in this respect, more like facts about the evaporation of water. They may sometimes be caused by social facts, but this is irrelevant when it comes to understanding what they are.

Social constructivism is widespread; in some circles, it may even be regarded as trivially true. So, why bother? The aim of this paper is not to *defend* or *argue against* social constructivism; it aims at a clarification of what social constructivism about mental disorder consists in, or *may* consist in. It will turn out that in the literature, one can find different versions of social constructivism. As these forms of social constructivism are often only implicit, part of the work will consist in uncovering some hidden constructivist commitments.

The paper proceeds as follows. The first section introduces some basic elements of social constructivism, including an elaboration on the difference between causal and non-causal, or, as I will also sometimes say, *metaphysical* explanation or explanatory dependence.^5^ The section “Versions of Social Constructivism about Psychiatry” sketches, in an abstract way, the various versions of social constructivism about psychiatry, and introduces the different targets of constructivist claims, such as mental disorders, their symptoms, and the property of being a mental disorder. Each of the remaining Sections [“Social Constructivism about Mental Disorders”, “Social Context, Experience, Phenomenology, and Symptoms”, and “Mental Disorder and Social Norms”] deals with a particular version of social constructivism about psychiatry. Some consequences for psychiatric explanation and, thus, for psychiatry as a science are highlighted in the conclusion. Note that the conclusions drawn in this paper are somewhat preliminary, in three respects. First, the way I will present the different explications of social constructivism is non-committal as to the metaphysical details of social constructivism. From the perspective of metaphysics, this may appear dissatisfying. But in order to pave the way for a more thorough theory of social constructivism in psychiatry, we should remain neutral on some of the metaphysical details. Second, the conclusions drawn below are preliminary in that the distinctions discussed here may not exhaust the field. I have focused on versions of social constructivism that appear to surface within prominent areas in philosophy and psychiatry. And although the taxonomy offered here is inspired by systematic considerations about candidate versions of social constructivism, I will not offer an argument to the effect that the versions of social constructivism discussed below exhaust the field. Finally, each of the versions discussed below deserves further attention. The paper offers partial explications of versions of social constructivism. Some aspects will be left out.

### Basic Tenets of Social Constructivism

Social constructivism about psychiatry is opposed to radical objectivism. Both claims concern the subject matter of psychiatry, the types of objects and the connections psychiatry deals with. The present section introduces the conceptual tools of social constructivism.

A quick look at social constructivism in other areas of philosophy will offer a more thorough idea of what social constructivism about some subject matter consists in. A prominent example that has been extensively discussed in the past years (8–11) is social constructivism about institutions or institutional facts. Social constructivism about institutions holds that institutions (or institutional facts) depend on specific intentional states of individuals. For instance, on this view, the fact that some sea-shell, dollar-bill, or coin is money depends on the fact that people collectively accept it as money [famously argued by Searle (8, 9)]. This form of social constructivism is characterized by its *target* (facts about social institutions, such as money), the relevant facts upon which its target is supposed to depend, or the target’s *social grounds* (in this case: facts about collective acceptance), and the *relation* that is supposed to hold between the two – in this case: some form of *metaphysical dependence*.

Here is the second example. Feminist philosophers have suggested that in some sense, gender is socially constructed (12, 13). On one version of this thesis, the idea is that women and men *become women* and *men* (in at least one significant sense of these terms) not due to their hard-wired biological make-up, but rather due to social causes, such as among other things, shared expectations on the side of caregivers, discursive practices (repeatedly marking the distinction between boys and girls/men and women), and esthetic practices within a society. Again, this form of social constructivism can be characterized in terms of its target (the occurrence of gender identities in individuals), the alleged grounds of this target (causal influences, such as expectations and discursive practices), and the connection between the two – here, *causation*.

Versions of social constructivism about psychiatry can be characterized in a similar format – in terms of their *target*, the alleged *grounds* of the target (I use “ground” for both, causal and non-causal grounds), and the *relation* that is supposed to hold between the two. We need to distinguish between two types of dependence relations – causal and non-causal dependence. As the reader might not be familiar with this distinction, let me illustrate the difference by way of some examples. An avalanche that occurs due to an earthquake is *caused* by the latter – and the avalanche can be causally explained by reference to the earthquake. When a rock hits a window so that the window shatters, the fact that the window shatters can be causally explained by reference to the fact that to rock hit the window. And when the heating of water leads to a transition from liquid to vapor, then the vaporization of water can be causally explained in terms of the heating of water. Causal explanations involve a temporal component – causes precede their effects. By contrast, the objects involved in *non-causal* explanations do not necessarily stand in a temporal successor relation. The existence of a forest depends on the existence of trees, and that there is a forest can be explained by reference to the presence of trees. But the existence of the trees does not cause the forest to exist, and the existence of the trees need not precede the existence of the forest. The hole in the Swiss cheese depends on the cheese; and the existence of the hole can be explained by reference to features of the cheese. But it need not be the case that there was, first, the cheese and then the hole. On naturalistic accounts of the mind, the processing of visual information depends on particular physiological processes. These processes underlie the processing of visual information, and the latter can be explained in terms of the former. But the physiological processes do not cause the processing of visual information and need not precede it.

“Because”-statements typically express explanations. Some explanations are causal, others are not. When speaking of dependence in what follows I mean *explanatory* dependence. An intuitive understanding of the difference between causal and non-causal explanation along these lines, in terms of examples and based on the observation that causal explanation essentially involves a temporal component non-causal explanation does not require, is sufficient for our present purposes. In the present context, non-causal dependence will sometimes be referred to as “constitution.”

As already indicated, versions of social constructivism may, in general, differ with respect to the relation they postulate between the social grounds of their targets and these targets. Versions of social constructivism fall into two categories – those that credit social facts with a relevant causal role for the etiology of the given target and those according to which the target non-causally depends on social facts. These categories mirror, to some extent, the following distinction drawn by Sally Haslanger:

*Causal Construction*: Something is causally constructed iff social factors play a causal role in bringing it into existence or, to some substantial extent, in its being the way it is.

*Constitutive Construction*: Something is constitutively constructed iff in defining it we must make reference to social factors (13, p. 98).^6^

This distinction applies in the context of theorizing about psychiatry. Consider the claim that some mental disorders depend on social norms (we will turn back to this view below, Section “Mental Disorder and Social Norms”) – that reference to social norms is relevant in an explanation for why a patient suffers from a mental disorder. Social norms may have a causal impact on the development of disorders, and facts about social norms may ground, in the non-causal sense, facts about disorders.

To illustrate, consider Stier’s [(2), p. 28] interpretation of Wakefield’s critique of current diagnostic practices in the case of anxiety disorders. Wakefield explains the fact that “current criteria allow diagnosis when someone is, say, intensely anxious about public speaking in front of strangers” by reference to “American society’s high need for people who can engage in occupations that require communicating to large groups” [(14), p. 154]. Stier appears to suggest that considerations like these reveal that there is a “[normative] impact of society on the concept of mental disorder” [(2), 28f.]. Although I doubt that Wakefield’s considerations concerning anxiety disorders support any such view,^7^ let us assume that the diagnostic practices, based on a given cultural background, in fact, ground facts about anxiety disorders. In this spirit, one may come up with an explanation of the following type:

[1] She suffers from anxiety disorder because her behavior violates a specific social norm, or shared expectation concerning the ability to speak publicly in front of strangers.

Taken in isolation, this explanation has *two* interpretations, corresponding to the two versions of social constructivism, a causal and a non-causal one. On the former, norm-violation plays a causal role in the occurrence of the anxiety disorder; and it may, in this respect, be similar to the case of evaporation of water and the human causes of global warming. On the latter, a behavioral pattern *counts* as an instance of anxiety disorder (in part), *because* it is an instance of a norm-violation.

Consider the *causal* reading first. Assume that, for some reason, a subject develops a minor anxiety concerning a particular type of social situation, say, to deliver a speech in front of strangers, in a social context where this form of anxiety, and the behavioral patterns that go together with it, are conceived of as socially awkward, or at least as not fulfilling a shared expectation. Showing the relevant behavior (say, some form of avoidance behavior, or specific behavior while delivering a speech) constitutes a norm violation; people react to the norm violation, thereby enforcing the anxiety in the subject – to a degree that it becomes pathological. In this case, actual social feedback in response to norm violation may trigger a feeling of shame, which, in turn, may cause the person to experience distress, which, in turn, may cause further deviations from socially expected behavior up to a degree that makes the condition pathological. Here, violation of social norms plays a causal role for the etiology of the disorder. This can easily be seen once we note the temporal component involved in the underlying process: *first*, there was norm violation which caused a certain behavior in the audience. The behavior in the audience *then* caused further distress, which, *after some time* and repeated stressful experiences, resulted in the development of an anxiety disorder.

On the other interpretation, the explanation does not commit one to there being a *development from* norm-violation *to* mental disorder. This is the interpretation (Stier’s) Wakefield appears to have in mind, when claiming that there is a normative impact of society on the concept of a mental disorder (on my interpretation: that the social norms sometimes determine, in a conceptual or metaphysical sense, what is a disorder and what is not). On this interpretation, *norm-violation* (or *being disposed to violate certain norms*) and *suffering from anxiety disorder* occur synchronically. The former partly grounds the latter, or, put differently, the latter can be metaphysically explained in terms of the former. There are several ways in which one can cash out talk of metaphysical explanation. For instance, one may suggest that the truth of a proposition that a person has a mental disorder is explained by a truth about the violation of social norms. Or, alternatively, one may want to claim that the instantiation of the property of having a mental disorder metaphysically depends on the occurrence of norm-violations. We need not go into the details here. For our present purposes, suffice it to note that there are at least two interpretations of the explanation that a person suffers from a mental disorder due to some social facts, a causal and a non-causal one, and that whatever the correct explication of the non-causal interpretation is, it will render the explanation true without any implications concerning a possible causal (and, thus, temporal) connection between norm-violation and having a mental disorder. An understanding of the distinction between causal and metaphysical dependence along these lines is sufficiently precise for the goals of the present paper. Social constructivism may involve both, a causal and a non-causal claim concerning the relation between mental disorders and social facts. Although this is probably true of most scientific explanation, it is worth pointing out that these explanations are, of course, only *partial* explanations; the presence of the explanans phenomenon is not fully explained in terms of its social causes or some underlying social facts. Versions of constructivism we will be dealing with in what follows are claims about the *partial* social construction of mental disorders. Purely physiological, behavioral, or experiential aspects that are not themselves socially constructed may be required to offer a full explanation of the relevant phenomena of mental disorders.

Before we turn to the specific versions of social constructivism about psychiatry – shouldn’t we say bit more about what makes social constructivism about psychiatry *social*? Intuitively, what depends on shared attitudes (like money), what varies with cultural context (like the social status of, say, a widow), or what itself essentially depends on a social object (like the property of playing in the NBL), is, in a sense, itself a social object. As a social object, it requires, at some stage, a sort of construction. There are straightforward examples of socially constructed objects; but this does not mean that there is a straightforward characterization of what social constructivism consists in. Any (at least partly successful) attempt to deliver a general answer to the question of what social constructivism is would transcend the boundaries of the present paper; but a general answer is not required – the theses we will be concerned with here are committed to the social dimension of their targets (such as particular mental disorders) in a *straightforward* sense; and we will be able to relate, in passing, these cases to intuitive formulations of social constructivism. Short reflection on two versions, one might, at first sight, want to classify as versions of social constructivism will help to get a better understanding of what makes social constructivism about psychiatry a version of *social* constructivism, and it will reveal that not all constructivist rhetoric amounts to constructivism as opposed to objectivism.

### Versions of Social Constructivism about Psychiatry

Social constructivism about psychiatry, as introduced above in contrast with radical objectivism, is heavily underdetermined. It is underdetermined with respect to its *target*, it is underdetermined with respect to the target’s *grounds*, and it is underdetermined with respect to the *relation* allegedly holding between the target and the target’s grounds. In this section, I will introduce five candidate targets for social constructivism about mental disorder: mental disorders themselves, the system of symptoms, phenomenology, and experience associated with a mental disorder, the second order property of being a mental disorder, and articulation of experiences and interpretation of utterances in patient–clinician interaction. The latter two can be dismissed immediately – they invite constructivist rhetoric at best. The candidate targets will have an impact on the candidate grounds and the relevant relation supposedly holding between the two.

Consider a person who has been diagnosed with PTSD and is, based on this diagnosis, classified as suffering from a mental disorder. This will, on the side of the patient, involve (a) PTSD itself, with its specific history, including the etiology or the trigger of PTSD, (b) a specific subjective experience and, possibly, a specific phenomenology, and a set of symptoms that are indicative of PTSD. Note that depending on the view one adopts regarding the nature of mental disorders, these may ultimately collapse into one single target, if the phenomenology, the subjective experience and particular symptoms enter the individuating criteria for the disorder itself;^8^ but even if this were the case, talk about symptoms, phenomenology, experience, and the disorder would still be acceptable. Drawing the terminological distinction appears to be innocent.

Furthermore, the classification of PTSD as a mental disorder will involve (c) a specific aspect of PTSD in virtue of which it counts as a mental disorder (rather than, say, a stressful episode of minor importance) and becomes clinically relevant. Finally, being diagnosed with and treated for PTSD typically requires that the patient interact with a clinician. She will (d) express her experiences and inner perspective as well as report symptoms in a particular way. On the side of the clinician, (e) an interpretation of the observed and reported (verbal and non-verbal) behavior of the patient is required.

For each of these targets, one can subscribe to the view that it is shaped by social facts. We have thus identified five candidate targets for social constructivism, all of which may give rise to a form of social constructivism about psychiatry, or at least may go together with some constructivist rhetoric. And indeed, all of these can be found in the literature. Before we turn to the more promising candidates for serious versions of social constructivism, let me briefly comment on the last two alleged targets, and corresponding claims concerning the impact of culture and social environment. Little reflection will reveal that cultural influence on patient–clinician interaction is irrelevant in the context of social constructivism about psychiatry, properly construed.

The DSM 5 contains a section “Cultural Formulation,” a revised version of what had already been presented in the previous manual. Its goal is somewhat difficult to identify. It contains information on mental disorder in relation to, well, *anything culture*, so to speak, beautifully illustrated by the suggested “Overall cultural assessment”:

Summarize the implications of the components of the cultural formulation identified in earlier sections of the Outline for diagnosis and other clinically relevant issues or problems as well as appropriate management and treatment intervention. (DSM 5, 750)

Information gathered about cultural background – including religious background and, possibly, gender identity – should inform diagnosis, clinician–patient interaction, and intervention. One particular reason for an assessment of cultural background is that differences in cultural background may cause confusion and misunderstandings. So, the manual includes questions that aim, in particular, at a clarification of *cultural concepts* and *idioms of distress* (DSM 5, 758 ff.), *some* of which concern the way the individual or members of the group the individual belongs to verbalize a given experience. One contention is, in this context, that knowledge of sociocultural background may facilitate access to underlying conditions; the idea does not seem to be that cultural expression of a disorder forms an integral part of the disorder itself (we will turn back to this below, in Section “Social Context, Experience, Phenomenology, and Symptoms”).

Does the claim that clinicians should be sensitive the culture-specific articulations of the underlying disorder constitute a version of social constructivism, in any interesting sense? This does not seem to be the case. To use an idiom from the natural science: some of the *data* we gather may be difficult to interpret. In psychiatry, the data may be difficult to interpret because they, first, involve verbal reports, whose real or intended meaning may escape the interpreter; and, second, the interpreter may exhibit something like a cultural bias, which makes interpretation of data difficult, too. But once properly interpreted, the content of the knowledge we acquire need not be knowledge involving cultural facts, just because access to such knowledge required reflection on cultural background. Let me illustrate the point by way of a (fictional) example, building on what one may want to call an informed stereotype: assume you enter a shop in Berlin, run by locals, say, a bakery. You buy a roll and a cake, adding up to € 2.45. You offer a € 20 bill. It may very well happen that the clerk looks at you like you’ve insulted him, refuses to take the bill, rolls his eyes and says: “Damn, I don’t have any change left!” Interestingly, this really does not mean that he does not have any change left. It appears to be some form of culturally determined expression of what elsewhere would probably have been expressed by something like: “Excuse me, do you have small change?” To properly interpret the utterance, knowledge of the cultural background is required. But of course, this does not mean that the fact (that there is only little change left) is constituted by these social facts knowledge of which is required to interpret the data. Claiming that in order to properly interpret the utterance in this context you have to take cultural considerations into account, does not imply that the content of the knowledge you end up with (if you’re successful) is knowledge about social facts.

Analogously, the claim that cultural considerations should play a role in clinician-patient interaction has no impact on the subject matter of psychiatry. It does have an impact on the practice of psychiatry. Data may be culturally determined. But data are not what psychiatry is ultimately about. We are mainly interested in the commitments of current psychiatry (and parts of philosophy of psychiatry) with respect to the relation between psychiatry and the natural sciences. The key question is not whether cultural differences may pose difficulties in the *assessment* of whether or not an individual suffers from a particular mental disorder. The question is, rather, whether the subject matter of psychiatry involves social explanations in terms of social facts or causes.

A similar point could be made about the recent trend to take cultural considerations into account in the context of health management [also present in the “Cultural Formulation” in DSM 5, see, for instance, questions regarding “expectations for services” that may depend on the individual’s cultural background (p. 752)]. The Supplement *Mental Health: Culture, Race, and Ethnicity*, edited by he U.S. Department of Health and Human Services, calls attention to the fact that cultural or racial background may have a significant impact on access conditions to mental health institutions. Thus, reflection on cultural aspects is of utmost importance in the context of psychiatric practice. But this does not amount to social constructivism about psychiatry.

The following three sections will introduce explications of the thesis that social context and culture shape mental disorder, which amount to forms of social constructivism about psychiatry in a more demanding sense.

### Social Constructivism about Mental Disorders

As noted above, one may subscribe to social constructivism concerning mental disorders themselves, such as schizophrenia, PTSD, or dissociative identity disorder. Social constructivism about mental disorders comes in two radically different forms. According to one view, which appears to be a minority view (at best), facts about mental disorders are like facts about Searlean institutions – whether or not someone is, say, an alcoholic depends, in a non-causal sense, on attitudes of other people according to which that person is an alcoholic. Being regarded as an alcoholic makes one an alcoholic. According to the other view, at least some mental disorders are individuated by their social causes, so that their instantiation non-causally depends on their etiology. This makes these disorders social in nature, although it remains to be seen whether it amounts to social constructivism, properly construed. Let me briefly comment on the first view, and then turn, in more detail, to the second.

Constructivist claims in metaphysics often take the following form:

[2] F’s are F’s because they are considered/regarded to be/experienced as F’s.

A famous instance of this schema is Searle’s claim that “[m]oney is money because the actual participants in the institution regard it as money” [(9), p. 17]. Some formulations in statements about the nature of mental disorders allow for a similar reading. Pickering, for instance, suggests that:

The relevant features of alcoholism do not, contrary to what it demands, exist independently of the category into which alcoholism is placed [viz. illness, *RvR*]. [(17), p. 27].

One can interpret this claim as follows: some features of alcoholism depend on specific attitudes toward alcoholism within a social context – namely, that it is regarded as alcoholism [rather than a mere moral weakness (Pickering) or, alternatively, as “manly” behavior].^9^ In their critical discussion of social constructivism about mental disorders, Kendler et al. [(5), p. 1145] appear to interpret social constructivism in a similar way, associating it with Haack’s characterization of a kind that is not real, i.e., not “independent of how we believe it to be” [(18), 132]. I am not entirely sure whether anyone ever held some such belief; but it appears that self-declared objectivists often tend to credit constructivists with some such view (so that the opponent of Kendler et al. would turn out to be a straw-man). One may suspect that Foucault accepted this form of constructivism (19), although Foucault, in later years, explicitly subscribed to a form of causal constructivism that is based on causal effects of discursive practices.^10^ Be that as it may – on this version of social constructivism, the *targets* are categories of mental disorder, the *grounds* of the targets are attitudes or discursive practices that involve the psychiatric category itself and attribute it to certain people, and the relation is that of non-causal dependence.^11^

This version of constructivism is not the only form of social constructivism regarding mental disorders. Some mental disorders seem to be *typically* caused by particular social events. PTSD is *typically* caused by experiences such as incarceration as a prisoner of war, or traumatic experiences that often have a social dimension, such as interpersonal violence or sexual assault.

Being typically brought about by social causes alone does, of course, not amount to social constructivism. To see that, consider the question of whether it contradicts radical objectivism about psychiatry. The radical objectivist may hold that mental disorders can be caused by social events, as long as we need not cite the social dimension among the relevant causes in an appropriate explanation that enables us to fully understand the mental disorder we are dealing with. The evaporation of water may often be caused by social events. A large part of the evaporation of water is currently caused by global warming (or so let us assume for the sake of the example). Global warming, in turn, is caused by our joint actions. And if global warming has a significant impact on the natural evaporation of water on earth, then, currently, the evaporation of water on earth is *typically* caused by social facts. However, this does not amount to social constructivism about the current evaporation of water – the question of whether or not evaporation of water is brought about by social causes is irrelevant to our understanding of what evaporation of water consists in. Put differently: *evaporation of water due to global warming caused by social events* is not an interesting type in physics or chemistry (though it may be an interesting type in the context of politics). The radical objectivist about mental disorders will maintain that similarly, questions regarding the causes of a disorder are, or should be, irrelevant in psychiatry.

However, trauma- and stressor-related disorders are partly *characterized* in terms of their etiology. Consider the general characterization of these types of disorder, taken from the DSM 5:

Trauma and stressor-related disorders include disorders in which exposure to a traumatic or stressful event is listed explicitly as a diagnostic criterion. (DSM 5, 265)

If the diagnostic criteria enter the taxonomy of mental disorders, then there are mental disorders that are partly individuated by their etiology. The motivation for this relational characterization is straightforward. Different experiences cause different subtypes of trauma- or stressor-related disorders, and knowledge of the cause may bear on clinical decisions. Often, the traumatic or stressful event involves a *social* dimension. Typical examples include experience of war, torture, incarceration as a prisoner of war, or sexual abuse. DSM 5 states explicitly that “[t]he disorder may be especially severe or long-lasting when the stressor is interpersonal and intentional” (DSM 5, 275). Interpersonal and intentional stressors involve a social dimension – not because any intentional action is social, bur rather because any intentional action directed at another person – here: the patient – is social. Witnessing a death by accident of a close relative and experiencing a catastrophe such as an anaphylactic shock form the *only* exceptions in the list included in DSM 5, waking during surgery forms a borderline case (in this case, it is not clear whether the fact that surgeons appear to interfere intentionally with the patient’s body plays a relevant explanatory role).

So, the family of trauma- and stressor-related disorders involves types of disorders that are, in part, defined in terms of the types of causes that actually triggered the disorder. PTSD due to traumatic experience during incarceration as a prisoner of war is different from PTSD due to traumatic experience caused by witnessing an accident involving the loss of a close relative. The difference shows at the token level, and it also shows at the level of subtypes, subtypes of the type *trauma or stressor-related disorder*, when the type of cause is referred to in a classification of the relevant disorder.

But doesn’t such relational individuation of types seem odd? Upon reflection, relational classifications are common. On most accounts, *being an artwork* depends on the etiology of the artwork – namely, being intentionally produced. Similarly, being an artifact depends on the etiology of the artifact. And individual horses are, on some views, horses in part because they are descendants from horses. In the social sciences, relational types are common: someone is a president (and not merely, say, a warlord), a state (and not merely a territory), or a law (and not merely an enforced rule) only if it has a certain history.

Thus, on the view implicit in DSM 5, for some subtypes of trauma- or stressor-related disorders, if a person suffers from this subtype, she does so because of the specific etiology of the disorder. The fact that the disorder has a cause of a particular type grounds the fact that it is a disorder of the corresponding subtype (for instance, *disorder caused by an interpersonal and intentional stressor*, or *PTSD due to torture*). Expressed in terms of targets, grounds, and the alleged relation between the two: the *targets* are subtypes of trauma- and stressor-related disorders. The grounds are complex events that relevantly involve a social dimension, such as intentions directed at other persons, interpersonal actions, and social properties, such as being a war and being an incarceration, which, moreover, stand in a causal relation to the psychological condition classified as a disorder. The relation between the social grounds and the occurrences of the disorder is, thus, causal. But the relation between the disorder and the cause is not *merely* causal, it is also conceptual, or metaphysical: to instantiate one of the subtypes requires that the disorder be caused by a particular type of object. The cause not only causes the disorder, facts about the cause also ground facts about the instantiation of the disorder. This marks the difference between the current evaporation of water, on the one hand, and the relevant types of mental disorders, on the other. Whereas, in fact, some events are evaporations of water and can be explained in terms of social events, causal explanations referring to social events are not required for an explanation of why evaporation of water is evaporation of water. Suffering from PTSD due to experience of interpersonal violence, in contrast, requires a specific causal history involving a social cause.

On this interpretation, some mental disorders are clearly *social objects* in the sense that they essentially involve a social aspect. But does this amount to social *constructivism*, in a straightforward sense? It fits Haslanger’s characterization, since in defining the subtype “we must make reference to social factors” [(13), p. 98]. The classification scheme involves types that are individuated by, and whose instantiation depends on, the presence of specific social features of the causes of the disorder. In some sense, however, this does not amount to social *constructivism*: it is not the case that forms of PTSD exist in or require for their existence, as Pickering put it, “a cultural or social frame of reference” [(17), p. 98]. Not every social object, such as abusive behavior, or incarceration, is a social construction in this sense. This may speak against Haslanger’s characterization or at least require elaboration on the notion of a social factor. Ultimately, this may be a verbal issue about how we want to use the term “constructivism”; what is important in the present context is that this type of classification of subtypes of disorders appears to contradict *objectivism*; in the present context, we may, then, group it with more demanding versions of social constructivism, discussed in the next sections.^12^

### Social Context, Experience, Phenomenology, and Symptoms

Let us move from aspects of social construction in classification to aspects of social construction involved in experience, phenomenology, and symptoms of mental disorders. Whether constructivism about experience, phenomenology, or symptoms is compatible with robust objectivism depends on whether we take these to be constitutive of the disorder.

As we have just seen, the claim that causes of mental disorders exhibit social features alone does not amount to any interesting form of social constructivism; a claim about *metaphysical* dependence on social causes was required. This should not come as a surprise. Recall the example discussed in Section “Basic Tenets of Social Constructivism”:

[1] She suffers from anxiety disorder because her behavior violates a specific social norm, or shared expectation concerning the ability to speak publicly in front of strangers.

On its causal interpretation, according to which causal norms played a causal role in the development of the disorder, committing to [1] one does not commit to any form of social constructivism. The objectivist may maintain that disorders have social causes; and the objectivist may consistently hold that *cultural variation among disorders depends on social causes*.

As already mentioned, DSM 5 offers a guide for cultural evaluations in the “Cultural Formulation,” which deals, to a significant extent, with problems in the interpretation of verbal reports; but it also deals with interpretation of experiences, calling attention to the fact that “the cultural constructs … influence how the individual experiences […] his or her symptoms or problems […]” (DSM 5, 750). As long as the subject’s reports are distinct from the underlying disorder, the claim that an adequate interpretation of reports requires knowledge about sociocultural background does not amount to social constructivism. The same holds for the claim that sociocultural background shapes the experience, phenomenology, and symptoms of the disorder, as long as these are not constitutive of the disorder itself, but, rather, *signs* of the disorder. On this picture, experiences, symptoms, and phenomenology may need translation just like reports. But signs of a disorder need not play an essential role in scientific classification. As long as the disorder manifests at the physiological level, and as long as types of disorders can, at least in principle, be individuated in purely physiological terms, the objectivist can happily admit that social causes have an impact on mental disorders [see, for instance, Ref. (5)]. The radical objectivist may even accept that knowledge of cultural context is epistemically relevant for clinical practice.

From the objectivist perspective, the connection between social causes and the way a disorder manifests may involve causal connections at the level of neurodevelopment. Kirmayer and Crafa (22) have, in the spirit of social or cultural neuroscience, argued that the physiological structure underlying specific conditions is shaped by social causes:

Culture can be seen as providing essential contexts for the development and functioning of the brain on multiple timescales: through its evolutionary history, which has involved brain–culture coevolution; across individual lifespans as biographical events are inscribed in circuitry by mechanisms of epigenetics and learning; and through ongoing influences on neural functioning by specific contexts of adaptation and performance. [(22), p. 7]

Again, this appears to be perfectly compatible with an objectivist stance – there is no social construction involved, if Kirmayer, Crafa, and others are right. Of course, these claims raise interesting methodological issues; for instance, if sociocultural background has a significant impact on the development of the physiology underlying mental disorders, can we dispense with descriptions in terms of sociocultural background, or does it provide heuristics that, for the time being, are indispensable in clinical contexts? To repeat: epistemic questions of this sort may arise even if we adopt objectivism about mental disorders. Unless we individuate disorders with respect to sociocultural causes, as suggested in DSM 5 for PTSD, we do not end up with a form of social constructivism.

If we do, however, we would end up with a very similar form of constructivism [as (Reviewer 2) has pointed out]: one may adopt the view that experiences, symptoms, or phenomenology are constitutive of a disorder, and that experiences, some symptoms and the phenomenology that goes together with a disorder are not individuated by their underlying physiology, but, rather, require individuation in terms of their social dimension [see Ref. (23–25) for a critical perspective]. Thornton (26) has recently pointed out that Jaspers (27), in his discussion of psychiatry (for instance, in his book “Allgemeine Psychopathologie,” first published in 1913), suggested that for a great number of psychological conditions that count as a mental disorder, some form of subjective understanding is required, besides observing behavior and understanding the other as rational (if possible), for an understanding of the kind of disorder the patient suffers from. Thornton tentatively agrees that an understanding of phenomenal aspects and subjective experiences may be required in understanding the nature of the disorder.

In a somewhat similar spirit, certain experiences may be regarded as forming essential parts of a disorder. Consider the following example, taken from Stier’s discussion of cultural variation in psychiatry:^13^

A […] striking cultural difference can be found in the case of social anxiety. While in the western cultural sphere this is connected with the fear of being harmed or offended, in Japan and Korea people are in fear of harming or offending others […]. [(2), p. 29]

Now, none of these claims makes, all by itself, for *social* constructivism; highlighting the way a disorder is presented to a subject from the first person perspective need not go together with social constructivism (although it will of course pose difficulties for forms of naturalism that typically go together with the form of objectivism we are concerned with here). However, the way a condition is presented to a subject from the first person perspective may *involve* a social dimension. Consider the case of variation across cultures in anxiety disorders: the assumption is that variation should be further explained in terms of cultural background, the role an individual is supposed to play within a community, the notion of personhood, etc. If we assume that the different forms of experience in western and Japanese and Korean culture correspond to at least two different subtypes of some disorder, subtypes any psychiatric taxonomy should be sensitive to, and if we assume that there is no unified physiological type corresponding to these experiences, we end up with a robust form of social constructivism. In Haslanger’s words: the subtypes are “causally constructed” in that “social factors play a causal role in bringing [them] into existence or, to some substantial extent, in [their] being the way [they are]” [(13), p. 98]. Our objectivist would oppose a view according to which genuine social experiences that are not individuated by their physiological basis are essential to classification in psychiatry. The targets are disorders, and the grounds are experiences, phenomenology, or symptoms that are individuated only with respect to a social context. The relation is, again, non-causal; the perspective of the individual is not causally connected to the disorder; it is constitutive of it.^14^

So, does *no* thesis regarding the social cause of mental disorders, all by itself, amount to social constructivism? Is all social construction non-causal, as far as psychiatry is concerned?

Consider one particular version of the claim that some disorders have (maybe among others) social causes. It is somewhat atypical. Standard versions of social constructivism regarding causal influences of the cultural background on phenomenology, symptoms, or experiences have it that cultural background does not contain any particular “information” on the disorder itself, which would explain cultural variation. For instance, the difference between social anxiety in Europe and some parts of East Asia is explained in terms of general differences regarding the concept of a person or the expectations regarding the relation between an individual and the community it lives in. In contrast, Ian Hacking, and, from a psychiatric perspective, Piper and Merskey (28) have argued that some mental disorders evolve in response to specific theories of disorders, and the expectations that go together with specific theories. Hacking writes:

[T]here was usually only one well-defined alter; today, sixteen alters is the norm. In France, a century or so ago, cases of doubling had the symptoms then associated with florid hysteria – partial paralyses, partial anesthesia, intestinal bleeding, restricted field of vision. English cases of double consciousness were more restrained but regularly went into a trance […]

Times change, and so do people. People in trouble are not more constant than anyone else. But there is more to the change in the lifestyle of multiples than the passage of time. We tend to behave in ways that are expected of us, especially by authority figures – doctors, for example. Some physicians had multiples among their patients in the 1840s, but their picture of the disorder was very different because the doctors’ expectations were different. That is an example of a very general phenomenon: the looping effect of human kinds. [(29), p. 21]

Hacking’s view is clearly not that there is one type of disorder that can be expressed or even experienced in different ways due to the different models of the disorder offered in a given social environment (such as *first* multiple personality disorder, *then* dissociative disorder). Rather, the idea is that the discourse in psychiatry itself has a causal impact on the disorder the patient develops. The discourse causes the individual to adopt the dissociative identity disorder personality. The discourse, including expectations and specific norms, functions as an external cause of the disorder itself.

Some remarks on the details of Hacking’s account are required in order to avoid misunderstandings. Basically, Hacking distinguishes between two different types of kinds, *indifferent* and *interactive kinds*. The former are kin to natural kinds; their objects are indifferent to our classification. Interactive kinds, however, involve causal feedback mechanisms, where the subjects that fall into the extension of an interactive kind respond to the classification, which, in turn, bears on the classification itself. This sort of causal looping effect is visible, according to Hacking, in psychiatric classification. But psychiatric classification is not purely interactive. According to Hacking, mental disorders have a physiological basis, which can be classified in terms of an indifferent kind. For what follows, I will be solely concerned with Hacking’s characterization of the interactive aspect of psychiatric classification.

The target is, again, a particular type of disorder. The grounds are social events, involving interaction between patient and clinician or members of the social environment, with certain individual or shared expectations. The dependence relation is causal – the person is caused to adopt a certain pattern of experiences and behavioral traits, which, in turn, may have an impact on the classification. In contrast with the versions of causal constructivism about mental disorders previously discussed, Hacking assumes that the very attribution of a disorder contributes to the disorder (or the interactive aspect of the disorder). Interestingly, this renders true an explanation that looks, at its surface level, very much like Searle’s constructivist claim about money:

[3] Some people suffer from dissociative identity disorder because others regard them as suffering from dissociative identity disorder.

Unlike Searle’s claim about money, however, [3] can be interpreted causally (and it is merely a partial explanation). It appears that there are causal versions of social constructivism in psychiatry. It is a form of constructivism not because of cultural variation, or because the disorder relates to attitudes like money relates to acceptance as money; it is a form of constructivism because it credits a conceptual practice, or discourse (that of multiple dissociative disorder) with the ability to literally create its own objects; and it does so in a way that masks the actual mechanisms that underlie the occurrence of multiple dissociative disorder. Of course, it is similar to cases where cultural background shapes the symptoms; discourse about multiple dissociative disorder is part of the cultural background. And if it has an impact on the development of dissociative disorders, including behavioral patterns, experiences, and symptoms, then in this case, cultural background shapes the disorder. But in this particular case, there is more: due to the tight connection between cultural background and disorder (i.e., the content of the discourse, which determines what it is to suffer from multiple dissociative disorder, and the disorder itself) the causal looping effect, if it occurs, ensures that that the current psychological condition instantiates the (first order) properties that define the interactive aspect of the disorder. Ignoring feedback mechanisms: The discourse causally contributes to the fact that the subject adopts the behavioral pattern others expect the subject to show; and showing the pattern is one mark of the disorder. If the interactive aspect of psychiatric classification were constitutive for facts about a subject falling under the relevant category,^15^ then the instantiation of the property of having dissociative identity disorder would metaphysically depend on facts about adaptation to expectations (among other facts). In this case, causal construction and metaphysical dependence go together.

Now, finally, let me turn to what may be regarded as the most common, and, at the same time, the most challenging version of social constructivism regarding psychiatry: the thesis that the property of *being a mental disorder* is itself socially constructed.

### Mental Disorder and Social Norms

In the tentative characterization of the notion of a mental disorder published in the introductory parts of DSM 5, the authors state that “[a]n expectable or culturally approved response to a common stressor […] is not a mental disorder” (DSM 5, 20). It seems that thereby, the authors intend to indicate that something is a mental disorder *only if* it involves responses to stressors that are *not* expectable or culturally approved. This reading is supported by the following passage:

The boundaries between normality and pathology vary across cultures for specific types of behaviors. Thresholds of tolerance for specific symptoms or behaviors differ across cultures, social settings, and families. Hence, the level at which an experience becomes problematic or pathological will differ. The judgment that a given behavior is abnormal and requires clinical attention depends on cultural norms that are internalized by the individual and applied by others around them, including family members and clinicians (DSM V, 14)

In contrast with the issues raised under the heading “Cultural Formulation,” this is clearly more than a purely epistemological point about access conditions. The authors suggest that whether or not a problematic experience is pathological depends in part on the social or cultural context. This idea has played a prominent role in the anti-psychiatrist movement (31), and the question of whether normative aspects involved in diagnostic procedures are social or can be cashed out in, say, descriptive or biological terminology has attracted considerable attention (3, 4, 26, 32). Being a mental disorder is a second order property, in the sense that it is supposed to be instantiated by first order properties of psychological conditions. An alcoholic exemplifies a specific psychological property, which, in turn, is supposed to exemplify the property of being a mental disorder.^16^ The significance of the property of *being a mental disorder* appears to stem from the fact that it distinguishes the clinically relevant from the clinically irrelevant (just like related concepts of health and illness). Social constructivism about this second order property states that whether or not it is exemplified by a first order psychological condition depends on the social norms within a society. The boundaries between the pathological and the non-pathological determine whether or not a condition is a mental disorder, and they are themselves partly determined by social norms. Very roughly, social norms are usually regarded as a particular type of expectations about the behavior of others (33, 34); individuals are expected to follow a norm and are expected to act in a certain way when they detect norm-violation. Only if some such structure is widespread among a society, a social norm is in place. Facts about social norms are clearly social facts. Consequently, this form of social constructivism maintains that facts about the second order property *being a mental disorder* (the target of this form of social constructivism) depend on social facts, such as the presence of social norms, and that the relation between the two is non-causal dependence – the social norms do not cause a condition to be a mental disorder; they determine, in a conceptual, or logical sense whether or not some condition counts as a mental disorder.^17^ Here, in a straightforward sense, instantiation of the second order property of being a mental disorder requires a certain conceptual framework; and it is shared attitudes that ground the instantiation of the property.

## Conclusion

Let me sum up the results. Versions of social constructivism come in three different forms. Their targets are (some or all) types of mental disorders, or the second order property of being a mental disorder. Social facts and events appear among the grounds of mental disorders, and social norms or expectations shape the boundaries of the second order property of being a mental disorder. It may even be the case that sometimes, psychiatric classification itself has an impact on the occurrence of its objects, as Hacking suggests. Note that apart from versions of social constructivism about the second order property of being a mental disorder, social constructivism about psychiatry may, and probably will be limited to *some* forms of mental disorders. What is true of trauma or stressor-related disorders need not be true of schizophrenia, or, in particular, neurodevelopmental disorders.

So, what’s the consequence of social constructivism for psychiatry as a science? It is difficult to give a straightforward answer. Quite a bit will hinge on general considerations about reduction, about the nature of scientific kinds, and on the nature of explanation. In order to bypass these further questions, let me begin with an observation that immediately follows from the different versions of social constructivism described above: if social constructivism about psychiatry is true, in one of the versions described above, then the conceptual apparatus psychiatry employs involves concepts of social objects, events, or facts. Moreover, it credits the so-represented events, objects, or facts with an explanatory role, in causal as well as metaphysical respects. Causal explanations in terms of social causes would turn out to be genuine and indispensable explanations within psychiatry – indispensable in the sense that the psychiatric types are partly social in nature. Although the radical objectivist may accept that some causal explanations of the occurrences of mental disorders that cite social events as causes are true, or may even play a useful heuristic role, she will deny that such explanations figure among the set of *psychiatric* explanations, properly construed. Recall: the evaporation of water from the Ocean may be caused by global warming, which, in turn, may be caused by social events. But an explanation of the evaporation of water from the Ocean based on its social causes will not count as an explanation within physics. Similarly, on the objectivist view, the explanation of mental disorders in terms of social causes will not count as a psychiatric explanation. Social constructivism, in some of its versions, is bound to deny that. Parts of psychiatry would then move toward the social sciences, as far as their explanatory practices are concerned.

Now, there is no consensus as to how we should conceive of scientific explanation, especially in the social sciences. One may take a liberal stance, suggesting that type construction and explanation mainly serve heuristic purposes, that causal generalizations merely systematize observations, and that consequently, the metaphor of the sociocultural environment *shaping* mental disorders can be given a non-committal, pragmatic, or purely epistemic interpretation. If so, the commitments of social constructivism are relatively weak; and dispute between constructivism and objectivism would be a dispute not about the nature, but rather about the pragmatically adequate or epistemically beneficial conceptualization of mental disorders. Some such considerations may have played a role in the history of DSM – the question of where to invest time and money may, by and large, be decided on pragmatic grounds.

But there is a corresponding ontic distinction that seems to better fit the nature of the dispute between constructivists and objectivists. The constructivist raises the worry that objectivism will fail for principled reasons; it just does not get the metaphysics right and, hence, looking for mechanisms or genetic profiles alone will never deliver the desired results. Rather, we should take social background, etiology, and cultural formation into account. And the objectivist maintains that at best, considerations regarding the sociocultural environment may play a heuristic role, or constitute an obstacle in the appropriate translation from behavioral observation and the subjects’ reports to the language of the underlying neural mechanisms.

Social constructivism, on its metaphysical interpretation, may have ramifications that go beyond issues that pertain to the metaphysics of science in the narrow sense. Some versions of social constructivism, concerning, for instance, gender or race categories, have been proposed not as a challenging exercise in theoretical metaphysics, but rather with a critical intention. Very roughly, the critique, whatever the details, departs from the observation that what *seems* to be a distinction, or is typically regarded as a distinction grounded in natural properties, in reality is a distinction imposed on the world *by us*, to a relevant degree. If true, this may have significant political and ethical ramifications – whether a status is natural or social bears on normative considerations. Revealing the hidden social nature of an allegedly natural category may then constitute a first step in a critical enterprise [for an explication of the idea of how social constructivisms relates to the critique of current practices, cf. Ref. (35)]. Uncovering the social nature of gender categories is important not only for general metaphysical purposes but also, and primarily, for political reasons. The idea is that if what appears to be natural turns out to be social, evaluative, and political practice should change.

We cannot go into the details here; but one may expect that similar critical or ameliorative projects will be relevant in the philosophy of psychiatry whenever evaluations based on the classification of people as having a mental disorder hinge on an objectivist interpretation of the property of having a mental disorder, where, in fact, having a mental disorder depends on social facts. If social norms determine whether or not a psychological condition belongs to the category of having a mental disorder, and if being classified as having a mental disorder forms the basis of unjust treatment by others because these others mistakenly believe the property of having a mental disorder to be natural, or objective, then uncovering the social nature of the property of being a mental disorder may bear on social practice. This is precisely what Szasz intended.

Let me close with a related observation regarding the distinction between objectivists and constructivists in psychiatry. As (Reviewer 2) has stressed, variation in the occurrence of a type depending on sociocultural background (such as the occurrence of specific gender identities) is often regarded as an indication of the fact that the occurrence of this type depends on social facts; social constructivism has been the theory of choice to account for such facts. Recall Hacking’s theory about the construction of dissociative identity disorders. Cultural variation is, here, clearly an indicator for the dependence of dissociative identity disorder on social facts. It is an indicator of the latter; but does cultural variation alone support some form or another of social constructivism?

Above, I have suggested that cultural variation in mental disorders is compatible with the form of objectivism discussed here. Doesn’t this show that the way I use the term “objectivist,” and, hence, “constructivist,” is at odds with at least one important use of these terms in the general debate on social constructivism? Here, I can merely gesture at an answer. I think that there is a difference between the pair “constructivist”/“objectivist” in the context of debates about first order mental disorders and, say, in the field of gender theory. This is not an accident; it is mainly due to the fact that the type of objectivism opposed, for instance, by feminist constructivists just lacks a counterpart in the sphere of psychiatry, because gender categories differ from categories of (first order) mental disorders in one important respect. Simplifying a lot, conceptualization in terms of gender will typically go together with an implicit naturalist conception of gender properties (sometimes described as *essentialization*). Consequently, the gender-objectivist believes gender to be part of the *nature* of an individual; being a woman is supposed to be inborn, deviations from the dichotomy are regarded as, well, non-natural and, possibly, requiring intervention. These issues simply do not arise in current day psychiatry. Conceptualizing someone as suffering from a disorder does *not* suggest a conceptualization of the person as having this property by nature.

In the context of psychiatry, *naturalist* or *essentialist* objectivism typically concerns the *second order* property of being a mental disorder; objectivists claim that it is a natural or essential property of (first order) psychological conditions, whereas constructivists maintain that classification of first order psychological conditions as a mental disorder comprises an element of construction.

## Author Contributions

The author confirms being the sole contributor of this work and approved it for publication.

## Conflict of Interest Statement

The author declares that the research was conducted in the absence of any commercial or financial relationships that could be construed as a potential conflict of interest.

## Footnotes

^1^Throughout this paper, I will ignore the possible cultural impact on experiments in psychiatric research, for two reasons. First, I doubt that it requires special treatment, if constructivism about experiments in psychiatry poses a problem at all. If social environment significantly shapes experimentation in psychiatry, it will do so in other sciences as well, and for similar reasons. By contrast, the targets of social constructivism discussed in this paper are, prima facie, *special*. Second, I am not aware of any form of constructivist rhetoric about experimentation in psychiatry that is relevantly distinct from constructivist rhetoric that shows in descriptions of patient–clinician interaction.

^2^Social facts, whatever these are, are no less *objective*, in the sense of “*real*,” than non-social ones, on the view endorsed here. It is a distinction inside naturalism. Social facts are part of nature in that they depend on natural facts about individual brains. This is an articulation of my conviction – most of the points made in the paper are compatible with more liberal views.

^3^I use the term “types,” in line with the literature, to designate what is signified by predicates, not to designate predicates or concepts expressed by predicates.

^4^Some such view can be ascribed to Kendell (3) and Boorse (4), at least about the second order property of being a mental disorder, a similar view is defended by Kendler et al. (5), who admit that social aspects may have an impact on disorders, but who assume that mental disorders can be individuated in a way similar to types in the natural sciences.

^5^There is a vast literature on the metaphysics of these forms of dependence; for introductory texts, see Ref. (6); for recent work on metaphysical dependence and explanation, see, for instance, the papers published in Ref. (7).

^6^It is worth noting that the claim about causal construction should be interpreted as a claim about types, rather than tokens. To return to our example from the introduction: the actual evaporation of water in the ocean is caused by social factors, and the way it proceeds also hinges on these social causes; but evaporation as such is independent of social factors; its being actually caused by social factors is irrelevant when it comes to understanding what it consists in, namely, some form of phase state transition. By contrast, to understand what gender categories consists in, social factors that played a role in the causal development of gender identities are in fact relevant. Why this is so is a question that transcends the boundaries of the present paper; it is a general question regarding classification in the social sciences.

^7^Stier is, as it seems, not quite right when suggesting that Wakefield *thereby* supports the claim that “the cultural setup […] tends to dictate the boundary between the normal and the deviant on the basis of the expected values and virtues of its members” [(2), p. 28]. Rather, Wakefield’s observation supports the epistemic claim that the perceived boundaries between the normal and the deviant are dictated (or maybe better: partly influenced) by the cultural setup. Wakefield stresses that there is a difference between false diagnostic practices and “social phobia [which is] a real disorder in which people can sometimes not engage in the most routine social interaction” [(14), p. 154].

^8^Let me give just two examples of views according to which symptoms may enter the individuating criteria for psychiatric types. If you assume, with Wakefield (15), that disorders are harmful dysfunctions, and that whether or not a certain psychological condition is harmful or a dysfunction may depend on the symptoms it produces, then symptoms may enter the individuating criteria for psychiatric taxonomies. You will end up with a similar result if you subscribe to what Murphy calls the “neo-Krapelinian picture” according to which “mental illnesses are regularly co-occurring clusters of signs and symptoms that doubtless depend on physical processes but are not defined or classified in terms of those physical processes” (16).

^9^Pickering distances himself from a social constructivist interpretation of this view – he regards the sort of construction involved in the classification of a condition as a mental illness as non-social. On his conception of social construction, social constructivists are wedded to the idea that what is socially constructed “exists only in certain cultural or social frames of reference” [(17), p. 98].

^10^The relevant power relations involve, according to Foucault, not only attributive practices but also causal feedback, for instance, in what he called biopolitics (20).

^11^It appears that on this view, psychiatry, conceived of as a science that deals with mental disorders, would rest on a confusion. The objects of psychiatry would turn out to be mere chimeras; psychiatry would turn out to be like a version of jurisprudence whose self-declared goal was to improve the law by empirical interventions on the minds of judges, based on the false assumption that the role judges play in the juridical system is determined by features intrinsic to judges themselves, while recognizing that typical properties relevant in the legal system are social properties. Moreover, the view appears to be at odds with the view that *suffering* correlates with the presence of conditions typically classified as disorders, or better, as (Reviewer 2) suggested, with the assumption that different types of suffering correlate with different types of disorders; so, it seems that there is more to these conditions than classification or being regarded as having a mental disorder (although this may indeed constitute part of the problem).

^12^Although this is not the topic of the present paper, two possible objectivist responses immediately come to mind: Maybe it is not the fact that the cause has certain social features, but rather that it is *perceived as* having these features, which is relevant to the type of disorder that we are dealing with. So, if reference to social features of causes just roughly catches what really does the explanatory work – that the cause is *perceived* as being of a particular social type – social constructivism about these types of disorders appears to be mistaken. Still, it is not entirely clear how to cash out, and account for, the relevance of perceived social causes within the radical objectivist framework. Second, one could construe this form of social constructivism as a form of pragmatic constructivism. Maybe Psychiatry, in its current form, is not as good as it gets. The types current psychiatry deals with are pragmatically adequate. If, for instance, incarceration as a prisoner of war is reliably connected with showing a specific behavioral pattern and being responsive to specific treatment, then, given our current epistemic background, individuation of subtypes of PTSD with respect to the causes makes perfect sense – for purely pragmatic reasons [according to Beebee and Sabbarton-Leary (21) fulfillment of some such condition is sufficient to count as a scientific type]. On this view, the mechanism underlying the connection between particular causes and particular disorders is still missing. Once uncovered, reference to the social cause becomes idle. The view that some mental disorders should, at the present stage of development of psychiatry, be classified in terms of their social causes is, thus, compatible with versions of in-principle reductionism about mental disorders. As a consequence, one may have reservations to group pragmatic constructivism with more robust forms of constructivism.

^13^Stier uses this example to back up an argument against biologism; it is not entirely clear to me how this example may affect even purely epistemic forms of reductionism, unless we buy, as a premise, that for biologism to be true, there must be *one physiological condition underlying both disorders*. But this does not seem to be required for some form or another of biologism to be true.

^14^There is, of course, also a relevant causal connection between the phenomenology or experience and the social environment of the subject, but this does not appear to be constitutive for the disorder.

^15^Tsou (30) has argued that if Hacking is right, then a stable classification of mental disorders in terms of the indifferent “part” of the classification should be possible. Here, we are not so much concerned with what Hacking actually claims; rather, we can, based on the work of Hacking, identify one possible version of social constructivism about mental kinds, a version of social constructivism that captures the interactive “part” of psychiatric classification.

^16^In a derivative sense, individuals instantiate the property of having a mental disorder, namely, when they instantiate a first order property (such as alcoholism), which exemplifies the second order property of being a mental disorder.

^17^Although Wakefield departs from a biological notion of dysfunction, he arrives at a similar form of social constructivism. Wakefield characterizes the concept of a disorder in terms of a “condition [that] causes some harm or deprivation of benefit to the person as judged by the standards of the person’s culture” – what he calls the “value criterion” [(15), p. 385], which complements the biologically construed dysfunction condition for the presence of a mental disorder. Wakefield assumes that disorders require biological dysfunction, but that what counts as a normal or acceptable life depends on social or cultural context.

## References

[1] US Department of Health and Human Services, editor. Mental Health: Culture, Race, and Ethnicity – A Supplement to Mental Health: A Report of the Surgeon General. Rockville, MD: U.S. Department of Health and Human Services; Substance Abuse and Mental Health Services Administration; Center for Mental Health Services (2001).

[2] Stier M. Normative preconditions for the assessment of mental disorder. Front Psychol (2013) 4:611. 10.3389/fpsyg.2013.00611 24058357 PMC3766858

[3] Kendell R The concept of disease and its implications for psychiatry. Br J Psychiatry (1975) 127:305–15. 10.1192/bjp.127.4.305 1182384

[4] Boorse C On the distinction between disease and illness. Philos Public Aff (1975) 5:49–68.

[5] Kendler KS Zachar P Craver C. What kinds of things are psychiatric disorders? Psychol Med (2011) 41:1143–50. 10.1017/S0033291710001844 20860872

[6] Hoeltje M Schnieder B Steinberg A, editors. Varieties of Dependence: Ontological Dependence, Grounding, Supervenience, Response-Dependence. Philosophia Verlag (2013).

[7] Correia F Schnieder B, editors. Metaphysical Grounding. Understanding the Structure of Reality. Cambridge: Cambridge University Press (2012).

[8] Searle J The Construction of Social Reality. London: Penguin (1995).

[9] Searle J Making the Social World: The Structure of Human Civilization. Oxford: Oxford University Press (2010).

[10] Tuomela R The Philosophy of Sociality. Oxford: Oxford University Press (2007).

[11] Epstein B The Ant Trap. Rebuilding the Foundations of the Social Sciences. Oxford: Oxford University Press (2015).

[12] Millett K Sexual Politics. London: Granada Publishing (1971).

[13] Haslanger S Ontology and social construction. Philos Top (1995) 23:95–125. 10.5840/philtopics19952324

[14] Wakefield JC The concept of mental disorder: diagnostic implications of the harmful dysfunction analysis. World Psychiatry (2007) 6:149–56. 18188432 PMC2174594

[15] Wakefield JC The concept of mental disorder: on the boundary between biological facts and social values. Am Psychol (1992) 47:373–88. 1562108 10.1037//0003-066x.47.3.373

[16] Murphy D Philosophy of psychiatry. 2012 Edition In: Zalta EN, editor. The Stanford Encyclopedia of Philosophy. (2010). Available from: http://plato.stanford.edu/entries/psychiatry/

[17] Pickering N The Metaphor of Mental Illness. Oxford: Oxford University Press (2006).

[18] Haack S Defending Science – Within Reason: Between Scientism and Cynicism. Amherst, NY: Prometheus Books (2003).

[19] Foucault M Histoire de la folie à l’âge classique: Folie et déraison. Paris: Plon (1961).

[20] Foucault M Histoire de la Sexualité (vol. 1): La Volonté de Savoir. Paris: Gallimard (1976).

[21] Beebee H Sabbarton-Leary N Are psychiatric kinds ‘real’? Eur J Anal Philos (2010) 6:11–27.

[22] Kirmayer L Crafa D What kind of science for psychiatry? Front Hum Neurosci (2014) 8:435 10.3389/fnhum.2014.00435 25071499 PMC4092362

[23] Gallagher S Delusional realities. In: Broome M Bortolotti L, editors. Psychiatry as Cognitive Neuroscience: Philosophical Perspectives. Oxford: Oxford University Press (2009). p. 245–66.

[24] Ghaeme S. Feeling and time: the phenomenology of mood disorders, depressive realism, and existential psychotherapy. Schizophr Bull (2007) 33:122–30. 10.1093/schbul/sbl061 17122410 PMC2632297

[25] Murphy D Psychiatry in the Scientific Image. Cambridge: MIT Press (2006).

[26] Thornton T Essential Philosophy of Psychiatry. Oxford: Oxford University Press (2007).

[27] Jaspers K Allgemeine Psychopathologie. Für Studierende, Ärzte und Psychologen. Berlin: Springer (1913).

[28] Piper A Merskey H. The persistence of folly: a critical examination of dissociative identity disorder. Part I. The excesses of an improbable concept. Can J Psychiatry (2004) 49:592–600. 15503730 10.1177/070674370404900904

[29] Hacking I Rewriting the Soul. Princeton, NJ: Princeton University Press (1995).

[30] Tsou J Hacking on the looping effects of psychiatric classifications: what is an interactive and indifferent kind? Int Stud Philos Sci (2007) 21(3):329–44. 10.1080/02698590701589601

[31] Szasz T The Myth of Mental Illness. London: Paladin (1972).

[32] Fulford K Analytic philosophy, brain science and the concept of disorder. In: Bloch S Chodoff P Green SA, editors. Psychiatric Ethics. Oxford: Oxford University Press (1999). p. 161–92.

[33] Elster J The Cement of Society. Cambridge: Cambridge University Press (1989).

[34] Bicchieri C The Grammar of Society: The Nature and Dynamics of Social Norms. New York: Cambridge University Press (2006).

[35] Haslanger S Gender and race: (what) are they? (what) do we want them to be? Noûs (2000) 34:31–55.

